# Multi‐Omics Analysis Reveals Sex‐Specific Signatures for BCG Vaccine Efficacy

**DOI:** 10.1002/eji.70144

**Published:** 2026-02-12

**Authors:** Qiuyao Zhan, Liang Zhou, Jianbo Fu, Xun Jiang, Xuan Liu, Wenchao Li, Simone J.C.F.M. Moorlag, Valerie A.C.M. Koeken, L. Charlotte J. de Bree, Vera P. Mourits, Leo A.B. Joosten, Yang Li, Mihai G. Netea, Cheng‐Jian Xu

**Affiliations:** ^1^ Centre For Individualised Infection Medicine (CiiM) a Joint Venture Between the Helmholtz Centre For Infection Research (HZI) and Hannover Medical School (MHH) Hannover Germany; ^2^ TWINCORE a Joint Venture Between the Helmholtz‐Centre for Infection Research (HZI) and Hannover Medical School (MHH) Hannover Germany; ^3^ Department of Internal Medicine and Radboud Community For Infectious Diseases (RCI) Radboud University Medical Center Nijmegen the Netherlands; ^4^ Department of Medical Genetics Iuliu Hatieganu University of Medicine and Pharmacy Cluj‐Napoca Romania; ^5^ Department For Immunology and Metabolism Life and Medical Sciences Institute (LIMES). University of Bonn Bonn Germany

**Keywords:** baseline immune status, BCG vaccine efficacy, multi‐omics, sex‐specific, systems immunology

## Abstract

Vaccines are a cornerstone of global public health, but their efficacy can vary significantly among individuals. Bacille Calmette‐Guérin (BCG) vaccine against tuberculosis is one of the most used vaccines, and its efficacy is influenced by numerous factors, including sex, age, and geographical location. Systematic investigations using large‐scale multi‐omics analyses to dissect sex‐specific determinants of vaccine efficacy remain limited. To better understand this variability and improve vaccine efficacy, we analyzed multi‐omics data from a cohort of 321 healthy individuals vaccinated with BCG, integrating immune cell frequencies, single‐cell RNA sequencing, plasma proteins, metabolites, and DNA methylation profiles. Our findings revealed significant sex‐specific differences in immune pathways that contribute to BCG efficacy. In males, pre‐vaccination signatures were associated with a stronger pro‐inflammatory response, highlighting the importance of an innate‐driven immune response, while females exhibited enhanced antigen presentation pathways and adaptive immune responses. This study underscores the need for understanding individual baseline immune status in relation to biological sex, an approach that could represent a promising path to optimizing vaccine effectiveness.

## Graphical abstract

Sex‐specific pre‐vaccination immune landscapes associated with effective BCG vaccine responses. Male high responders exhibit a monocyte‐enriched, pro‐inflammatory innate immune profile, whereas female high responders display immune signatures associated with enhanced adaptive immune responses.

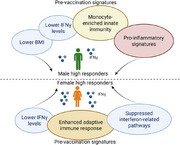

## 1 Introduction

Infectious diseases remain a major threat to global health, and vaccines are our most effective strategy to combat them. Among them, the Bacille Calmette–Guérin (BCG) vaccine is the only approved vaccine against tuberculosis (TB), caused by *Mycobacterium tuberculosis* (*Mtb*) [1]. With over 4 billion administrations worldwide, BCG remains the most widely used vaccine globally.

BCG vaccination has been shown to induce a broad upregulation of the cytokine production capacity of immune cells, enhancing the production of TNF, IL‐2, IL‐4, IL‐10, and IL‐17, alongside interferon‐gamma (IFN‐γ) [2]. IFN‐γ production is widely used as the primary marker of vaccine efficacy due to its central role in activating macrophages to kill *Mtb [* 1, 3] and establish adaptive immune memory. However, BCG's protective efficacy varies greatly among individuals, with rates ranging from 0% to 80% [4]. To enhance vaccine efficacy, it is essential to understand the underlying factors contributing to this variability in immune response.

Omics approaches have been widely employed to investigate the effects of BCG vaccination on epigenome [5], proteome [6], metabolome [7], microbiome [8], and single‐cell transcriptome [9]. While these approaches have provided valuable insights, it is difficult to capture the complex, interdependent interactions using a single‐omics approach [10, 11, 12]. A more comprehensive understanding requires integrating data from transcriptomics, proteomics, metabolomics, and other high‐throughput technologies.

Interestingly, the protective effects of vaccines, including BCG, are influenced by biological sex [13, 14, 15, 16, 17]. Studies have shown that BCG induces stronger protection in girls than in boys after 1 week [13]. Similarly, male mice display weaker protection against *Mtb* and reduced T‐cell responses after BCG vaccination, compared with female mice [18]. This variation is partly due to the influence of sex steroid hormones on immune responses, which is observed in both human and animal models [19, 20]. Previous systems immunology studies have explored mechanisms underlying immune sex differences, such as the study by David et al., [21]. However, large‐scale multi‐omics studies dissecting sex‐specific drivers of vaccine efficacy remain limited.

To address this gap, we utilized multi‐omics immunological profiling to systematically compare how host factors, including epigenomic, proteomic, metabolomic, flow cytometric, and single‐cell transcriptomic data, influence vaccine efficacy between sexes. Identifying pre‐vaccination signatures that predict antibody and cellular responses and understanding these underlying biological pathways could lead to more effective strategies for enhancing vaccine responses [22].

In this study, we employed a systems immunology approach to investigate sex‐specific differences in response to BCG vaccination using a cohort of 321 healthy individuals, equally distributed between males and females. Our analysis included immune cell frequencies, single‐cell RNA sequencing (sc‐RNAseq), circulating plasma proteins, metabolites, and DNA methylation. By integrating multi‐omics data, we aimed to identify sex‐specific signatures linked to vaccine efficacy, paving the way for more personalized and effective vaccination strategies.

## 2 Results

### 2.1 Females Exhibit Significantly Higher Vaccine Efficacy Compared with Males

**FIGURE 1:**
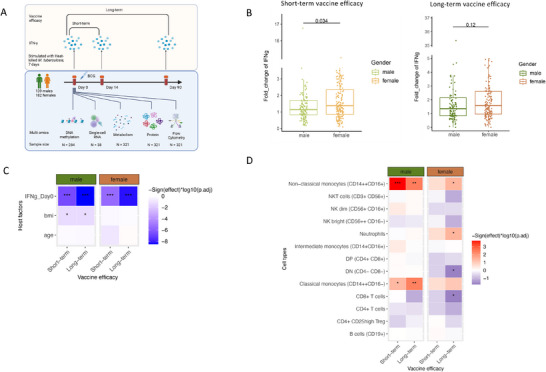
Individual host factors associated with vaccine efficacy. (A) Overview of the study design. A cohort of 321 healthy participants (139 males and 182 females) was sampled at Day 0 (pre‐vaccination), Day 14, and Day 90 after BCG vaccination. IFN‐γ production was measured after 7‐day stimulation with heat‐killed *M. tuberculosis*. Baseline multi‐omics data profiling, including DNA methylation (*N* = 284), single‐cell RNA sequencing (*N* = 38), metabolomics (*N* = 321), proteomics (*N* = 321), and flow cytometry (*N* = 321). (B) Boxplot showing the difference in vaccine efficacy at 14 days (short‐term vaccine efficacy, left) and 90 days (Long‐term vaccine efficacy, right) between males (green) and females (orange). The Wilcoxon rank‐sum test was used to assess the significance of the differences. (C) Heatmap showing the correlation between vaccine efficacy and host factors. Color intensity indicates the strength and direction of the correlation. (D) Heatmap showing the association between vaccine efficacy and immune cell frequencies. Correlation significance is indicated as follows: *p < 0.05, **p < 0.01, ***p < 0.001. Red bars represent the positive correlation, while blue bars represent negative correlations. The schematic figure was created by BioRender.com.

To assess interindividual variation in vaccine efficacy, we utilized the 300BCG cohort [6, 7, 9, 23], which consists of 321 healthy individuals measured at three‐time points: before the BCG vaccine (day 0), 14, and 90 days postvaccination. The overall study design is presented in Figure 1A, with age and sex equally distributed (Figure S1). Vaccine immunogenicity against tuberculosis was evaluated through immune profiling, focusing on cytokine production capacity in response to stimulation with heat‐killed *Mtb* H37Rv (5 µg/mL, 7‐day stimulation) from blood samples collected at each time point. The fold change of IFN‐γ concentrations in response to *M. tuberculosis* stimulation from baseline to day 14 (short‐term efficacy) and day 90 (long‐term efficacy) served as the primary measure of vaccine efficacy.

As illustrated in Figure 1B, female individuals exhibited significantly higher short‐term vaccine efficacy than males (*p* = 0.034, Wilcoxon test). This trend persisted in long‐term efficacy, although the difference was less pronounced (*p* = 0.12, Wilcoxon test). These results indicate sex‐based differences in vaccine efficacy, with females displaying a stronger IFN‐γ response following BCG vaccination [6, 18].

### 2.2 Individual Host Factors Associated with Vaccine Efficacy

Given the observed sex‐based differences in vaccine efficacy, we further investigated whether individual host factors might contribute to this variability. Correlations between vaccine immune responsiveness and host factors such as BMI, age, and pre‐vaccination IFN‐γ levels were analyzed separately for males and females. Interestingly, we observed that BMI was significantly negatively correlated with vaccine responses in males (short‐term: *r* = −0.21, p_FDR‐adjust_ = 0.034; long‐term: *r* = −0.19, *p*_FDR‐adjust_ = 0.042, Spearman correlation test), while no such correlation was present in females (short‐term: *r* = −0.02, *p*_FDR‐adjust_ = 0.97; long‐term: *r* = −0.002, *p*_FDR‐adjust_ = 0.97, Spearman correlation test, Figure 1C). Consistent with these sex‐stratified correlations, a unified regression model including a BMI × sex interaction while adjusting for age confirmed a significant sex difference in the BMI–efficacy association for the short‐term endpoint (interaction *p* = 0.046) and a similar, but weaker, trend for the long‐term endpoint (interaction *p* = 0.074). Moreover, baseline IFN‐γ production capacity was significantly negatively correlated with vaccine immunogenicity in both sexes (in males, short‐term: *r* = −0.48, *p*_FDR‐adjust_ = 6.53 × 10^−8^, long‐term: *r* = −0.55, p_FDR‐adjust_ < 2.2 × 10^−16^; in females, short‐term: *r* = −0.34, *p*_FDR‐adjust_ = 2.98 × 10^−5^, long‐term: *r* = −0.46, p_FDR‐adjust_ = 1.19 × 10^−8^, Spearman correlation test). This indicates that individuals with a higher pre‐vaccination IFN‐γ production capacity experienced a smaller increase in IFN‐γ production after vaccination.

To further elucidate contributors to the observed sex‐based differences, we examined associations between baseline immune cell frequencies and vaccine response. In males, the proportions of classical monocytes (CD14^++^ CD16^−^) and nonclassical monocytes (CD14^++^ CD16^+^) showed significant positive associations with both short‐term and long‐term vaccine responses (*p*_FDR‐adjust_ < 0.05). In females, similar trends were observed, but statistical significance was achieved only for long‐term immune responses (*p*_FDR‐adjust_ < 0.05). Notably, the male–female difference in the association for nonclassical monocytes was significant at the short‐term endpoint (interaction *p* = 0.026), indicating that the cell proportion and short‐term vaccine efficacy relationship differs by sex. Additionally, sex‐stratified analyses suggested CD8^+^ T cell proportions were negatively associated with long‐term vaccine efficacy in females (*p*_FDR‐adjusted_ < 0.05; Figure 1D). However, the corresponding sex‐specific difference was not statistically significant (interaction *p* = 0.34).

### 2.3 Baseline DNA Methylation Levels Associated with the BCG Vaccine Efficacy

**FIGURE 2:**
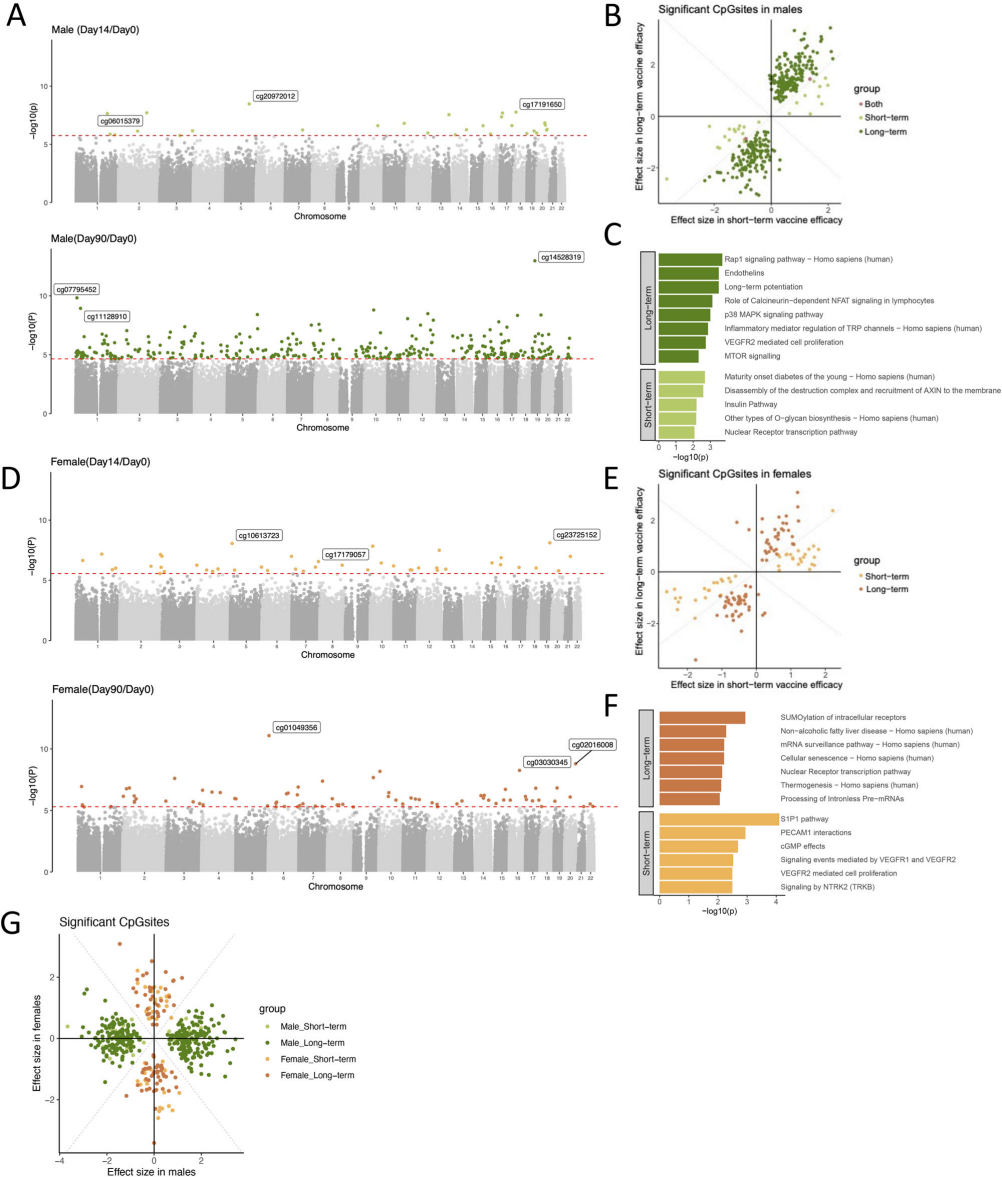
Baseline DNA methylation levels associated with the BCG vaccine efficacy. (A) Manhattan plots for the associations between short‐term (top) and long‐term (bottom) vaccine efficacy and baseline DNA methylation in males. The red line shows the genome‐wide significant threshold (*p*_FDR‐adjust_ < 0.05) for multiple testing. Methylation CpG sites that surpassed the *p*_FDR‐adjust_ threshold were highlighted in green. (B) Scatter plot showing the effect size of significant CpG sites in males (*p*_FDR‐adjust_ < 0.05). The *x*‐axis represents the effect size of CpG sites associated with short‐term vaccine efficacy, and the *y*‐axis represents the effect size of the CpG sites associated with long‐term vaccine efficacy. The data points are color‐coded based on their significance: Light green points for CpG sites significant for short‐term efficacy, dark green for those significant for long‐term efficacy, and orange for those significant for both vaccine efficacy. (C) Bar plot showing pathway enrichment for genes near the significant CpGsites in males and (F) females. Only pathways with *p*‐values less than 0.05 are shown. Each pathway includes at least three genes matched to the relevant pathway. (D) Manhattan plots for the associations between short‐term (top) and long‐term (bottom) vaccine efficacy and baseline DNA methylation in females. The red line shows the genome‐wide significant threshold (*p*_FDR‐adjust_ < 0.05) for multiple testing. Methylation CpG sites that surpassed the *p*_FDR‐adjust_ threshold were highlighted in orange. (E) Scatter plot showing the effect size of significant CpG sites in females (*p*_FDR‐adjust_ < 0.05). The data points are color‐coded based on their significance: light orange points for CpG sites significant for short‐term efficacy and orange for those significant for long‐term efficacy. (G) Scatter plot showing the effect size of significant CpGsites in males and females (*p*_FDR‐adjust_ < 0.05). The *z*‐axis indicates the effect size of CpGsites associated with vaccine efficacy in males, and the *y*‐axis indicates the effect size of the CpGsites associated with vaccine efficacy in females. The data points are color‐coded based on their significance: Light green points for CpG sites significant for short‐term efficacy, dark green for those significant for long‐term efficacy, and orange for those significant for both vaccine efficacy in males. Light orange points for CpG sites significant for short‐term efficacy, orange for those significant for long‐term efficacy in females.

Building on the observed sex‐based differences in immune characteristics, we next investigated the underlying regulatory mechanisms at the epigenetic level. Specifically, we analyzed the association between baseline DNA methylation and vaccine efficacy, focusing on sex‐specific patterns. In males, we identified 28 CpG sites genome‐wide significantly associated with short‐term vaccine efficacy and 334 CpG sites significantly associated with long‐term vaccine efficacy (*p*_FDR‐adjust_ < 0.05; Figure 2A). Notably, as shown in Figure 2B, 98% of these significant CpG sites were clustered in the first and third quadrants, indicating a consistent effect direction for these sites across both short‐term and long‐term vaccine efficacy. Among these CpG sites, the most significant was located in the *GIPC1* gene (cg14528319, *p*_FDR‐adjust_ = 8.00 × 10^−8^). *GIPC1* is known for its role in integrin recycling, which is critical for cell migration, angiogenesis, and cytokinesis [24]. This suggests that *GIPC1* may influence immune cell behavior relevant to vaccine response, particularly through pathways associated with cell adhesion and migration. Interestingly, some significant CpG sites were found near interleukin gene *IL13* (cg24580593) and *IL16* (cg03156543), suggesting potential epigenetic regulation that may influence the expression of these genes, particularly by influencing Th2‐driven inflammation for IL‐13 and immune cell recruitment via IL‐16. Pathway analyses of genes annotated to the significant CpG sites associated with long‐term vaccine efficacy revealed enrichment in cellular signaling pathways, including the vascular endothelial growth factor (VEGFR) signaling, Rap1 signaling, p38 mitogen‐activated protein kinase (MAPK) signaling, and mechanistic target of rapamycin (mTOR) signaling pathways (Figure 2C). In contrast, pathway analysis of genes associated with short‐term vaccine efficacy highlighted enrichment in the insulin pathway and O‐glycan biosynthesis.

Having characterized the epigenetic factors associated with vaccine efficacy in males, we next investigated whether similar regulatory patterns were present in females. In females, we detected 44 genome‐wide significant CpG sites associated with short‐term vaccine efficacy and 79 significant CpG sites associated with long‐term vaccine efficacy (*p*_FDR‐adjust_ < 0.05, Figure 2D). Interestingly, as shown in Figure 2E, the distribution of effect sizes in females indicates a consistent direction of effects for these sites across both short‐term and long‐term vaccine efficacy, similar to what we observed in males.

Pathway enrichment analysis in females highlighted genes annotated to these significant CpG sites as being involved in fundamental cellular processes such as transcription regulation, mRNA surveillance, and immune cell migration (Figure 2F).

After characterizing the regulatory methylation factors associated with vaccine efficacy in both sexes, we conducted a comparative analysis of effect sizes to directly assess differences between males and females. As illustrated in Figure 2G, the distribution of significant CpG sites highlights distinct sex‐specific patterns, with effect sizes clustering into separate groups for males and females. To quantify the separation observed in Figure 2G, we compared effect‐size magnitudes within each sex‐significant CpG set. For CpGs significant in males, effect sizes were consistently larger in males than in females (short‐term: median Δ_abs_ = −1.05, Wilcoxon *p*_FDR‐adjust_ = 3.7 × 10^−^^9^; dominance = 1.00, binomial *p*_FDR‐adjust_ = 3.7 × 10^−^^9^; long‐term: median Δ_abs_ = −1.19, Wilcoxon *p*_FDR‐adjust_ = 1.7×10^−^^5^ ^6^; dominance = 1.00, binomial *p*_FDR‐adjust_ = 3.7 × 10^−^^1^ ^0^ ^0^). Conversely, for CpGs significant in females, females showed systematically larger effect sizes (short‐term: median Δ_abs_ = +0.866, Wilcoxon p_FDR‐adjust_ = 4.6 × 10^−^^1^ ^3^; dominance = 0.977, binomial *p*_FDR‐adjust_ = 5.1 × 10^−^^1^ ^2^; long‐term: median Δ_abs_ = +0.933, Wilcoxon *p*_FDR‐adjust_ = 5.9 × 10^−^^1^ ^5^; dominance = 1.00, binomial *p*_FDR‐adjust_ = 2.2 × 10^−^^2^ ^4^). These results statistically confirm that male‐significant and female‐significant CpG sets follow distinct sex‐dominant effect‐size patterns.

### 2.4 Pre‐vaccination Inflammatory Profiles in Monocytes Modulate the Transcriptional Response to BCG Vaccination

**FIGURE 3:**
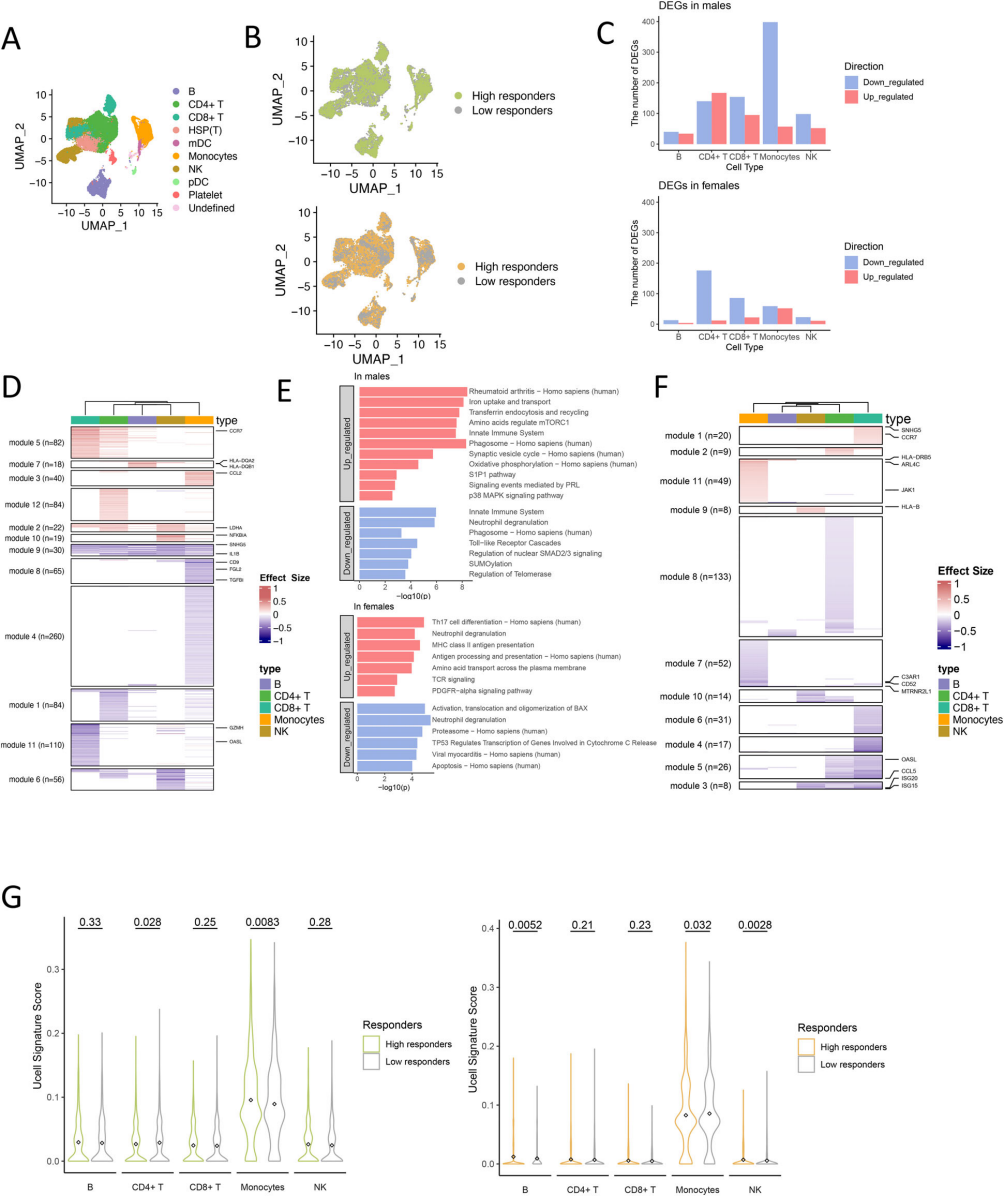
Pre‐vaccination inflammatory profiles in monocytes modulate the transcriptional response to BCG vaccination. (A) UMAP visualizes the cells from 38 individuals before vaccination. Cells (dots) are colored by annotated clusters. (B) UMAP shows cells colored by high responders and low responders in males (top) and females (bottom). (C) The number of differentially expressed genes (DEGs) colored by up‐ and downregulated in high responders compared with low responders in five main cell types. Blue bars represent the number of downregulated genes, and red bars represent the number of upregulated genes. (D) Fold changes of DEGs (rows) in five main cell types (columns), with insignificant values (*p*_FDR‐adjust_ > 0.05) set to 0. Genes were grouped into modules through unsupervised k‐means clustering in males and (F) females. (E) Top: Bar plot showing results of pathway enrichment for genes from module 3, and module 4 & module 8 in (D). Bottom: Bar plot showing results of pathway enrichment for genes from module 11 and module 7 in (F). Blue bars represent the number of downregulated pathways, and red bars represent the number of upregulated pathways. (G) Pre‐vaccination signatures in five main cell types in males (left) and females (right). The violin plots are color‐coded based on high and low responders. The diamond shape in the boxplot represents the mean value of the respective UCell scores. Wilcoxon's rank‐sum test was applied to compare the UCell scores between high responders and low responders.

To further investigate the cellular mechanisms underlying the observed sex‐specific associations with vaccine efficacy, we analyzed scRNA‐seq data at baseline. This dataset includes a total of 97,992 cells from 38 individuals, representing a subset of the 300BCG cohort [9]. Using Uniform manifold approximation and projection (UMAP), we visualized the clustering of eight main immune cells at baseline (Figure 3A). Individuals were stratified into high and low responders based on the median fold change in IFN‐γ production 14 days after BCG vaccination. One participant without Day 14 IFN‐γ data was excluded. This stratification included 7 high and 10 low responders among males and 13 high and 7 low responders among females. The proportions of high and low responders were comparable between females and males in the full cohort (Figure S2A). On the UMAP plot, cells from high and low responders were evenly distributed, without distinct clustering in either sex (Figure 3B). To assess whether the trends observed in this smaller scRNA‐seq subset (*n* = 37) aligned with those in the broader 300BCG cohort (*n* = 321), we compared the cell compositions between high and low responders. Dirichlet regression analysis revealed no statistically significant differences in cell proportions of each cell type between the two groups (Figure S2B).

We next performed a differential expression analysis on five major cell types: B cells, CD4^+^ T cells, CD8^+^ T cells, monocytes, and NK cells. This analysis identified a total of 1235 significant differentially expressed genes (DEGs) between high responders and low responders in males (*p*_FDR‐adjust_ <0.05, |avg_log2FC| > 0.1), with monocytes exhibiting the highest number of DEGs (Figure 3C). In females, 458 significant DEGs (*p*_FDR‐adjust_ < 0.05, |avg_log2FC| > 0.1) were identified. Notably, more DEGs were associated with CD4^+^ and CD8^+^ T cells in females.

To identify gene expression patterns associated with specific cell types, we conducted k‐means clustering of the DEGs, identifying 12 distinct modules in males (Figure 3D). Most modules exhibited strong cell‐type specificity. Notably, module 9 was broadly downregulated across multiple cell types and included *SNHG5*—a small nucleolar RNA host gene, whose reduced expression has been linked to decreased inflammatory responses [25]. In B cells, module 7 displayed upregulation of MHC class II genes, *HLA‐DQA2* and *HLA‐DQB1*, crucial for antigen presentation to CD4^+^ T helper cells, potentially enhancing immune activation [26].

Given the significant positive association between monocyte subsets and vaccine efficacy in males, as well as monocytes exhibiting the highest number of DEGs (*n* = 455) between high and low responders, we next focused our analysis on monocyte‐specific upregulated and downregulated modules. Pathway enrichment analysis of modules 3, 4, and 8 (monocyte‐specific) highlighted the involvement of mTOR, p38 MAPK signaling pathways, and Toll‐like receptor cascades (Figure 3E, top; Table S1). In addition, pathway‐level analysis from a complementary pseudobulk model identified significant enrichment of pathways such as the mTORC1, interleukin, and interferon signaling pathways, further supporting that these responder‐associated effects were stronger in males than in females (Table S2). These pathways align with the findings at the epigenetic level, which highlight the role of monocytes in shaping an inflammatory environment critical for effective vaccine responses [27, 28].

Expanding on our findings in males, in whom monocytes were linked to proinflammatory pathways associated with vaccine efficacy, we assessed whether a similar transcriptional profile exists in females. Through comparable analyses, we identified monocyte‐specific modules 11 and 7 in females (Figure 3F). Pathway enrichment revealed associations with antigen presentation and T helper cell differentiation, including Th17 cell differentiation, MHC class II antigen presentation, and TCR signaling. Additionally, downregulated pathways encompassed apoptotic processes and the TP53 regulatory pathway, suggesting suppression of cell death pathways mechanisms that may preserve monocyte function and longevity, supporting a sustained immune response (Figure 3E, bottom). These findings suggest that while monocytes play a role in vaccine efficacy across both sexes, the underlying immune pathways may differ, with females showing a more pronounced adaptive (antigen presentation) immune signal (Figure S2C).

Building on the observation that females exhibit a greater number of DEGs within T cells, and the association between CD8^+^ T cell proportions and vaccine efficacy, we further explored the functional enrichment of DEGs in female T cells (Figure S3A). Pathway enrichment analysis revealed a notable downregulation of interferon‐related signaling pathways, including IFN‐γ signaling. This aligns with our earlier finding of a negative correlation between baseline IFN‐γ production and vaccine efficacy in females. Conversely, in males, downregulated pathways in high responders included IL‐12‐mediated signaling and Th1 and Th2 cell differentiation, contrasting with the adaptive immune emphasis observed in females (Figure S3B). To further dissect sex‐specific transcriptional programs, we compared the CD8^+^ T‐cell effect sizes between males and females and identified modules exhibiting clear sex‐restricted patterns. This analysis confirmed that the IFN‐associated module (module 3) was selectively downregulated in females, indicating a female‐specific suppression of IFN‐related programs. Conversely, in males, high responders exhibited distinct transcriptional signatures not observed in females, including downregulation of module 1 (enriched for mitochondrial respiratory and TCR‐signaling pathways) and upregulation of module 7 (enriched for translation‐related pathways) (Figure S3C,D).

To validate the role of pre‐vaccination pro‐inflammatory response in male monocytes, we employed a previously established pro‐inflammatory gene set derived from a pan‐vaccine study [27] of 13 vaccine platforms. This gene set demonstrated that pre‐vaccination immune state in innate immune cells correlates with antibody response, though that study did not include BCG vaccination. Using this gene set, which encompasses 14 genes involved in pro‐inflammatory cytokine and chemokine responses (e.g., *CXCL10*/*IP‐10*, *CCL20*, mediators of *IL1*, NF‐κB signaling: *MAPK8IP1*, *CASP5*, and *EGR*, and NF‐κB target genes: *KCNJ2*, *PTGS2*, and *ZNF248*), we calculated a gene set activity score. Our analyses revealed that these 14 genes showed elevated activity in male monocytes compared with other cell types and were significantly upregulated in high responders (*p* = 0.0083, Wilcoxon test) (Figure 3G, left). Interestingly, in females, low responders displayed significantly higher activity of the same gene set in monocytes than high responders (*p* = 0.032, Wilcoxon test) (Figure 3G, right). These findings further support our conclusion that the enhanced vaccine efficacy observed in males is linked to the baseline pre‐vaccination pro‐inflammatory state within monocytes.

In addition, the module‐score analysis of the TP53 regulatory program in monocytes and IFN–γ signaling signatures in CD8^+^ T cells showed significantly high–low responder difference in females, whereas males displayed no significant difference between responder groups (Figure S3E‐F).

### 2.5 Protein and Metabolite Associations with Vaccine Efficacy Identify IL‐10 as a Key Marker in Females

**FIGURE 4:**
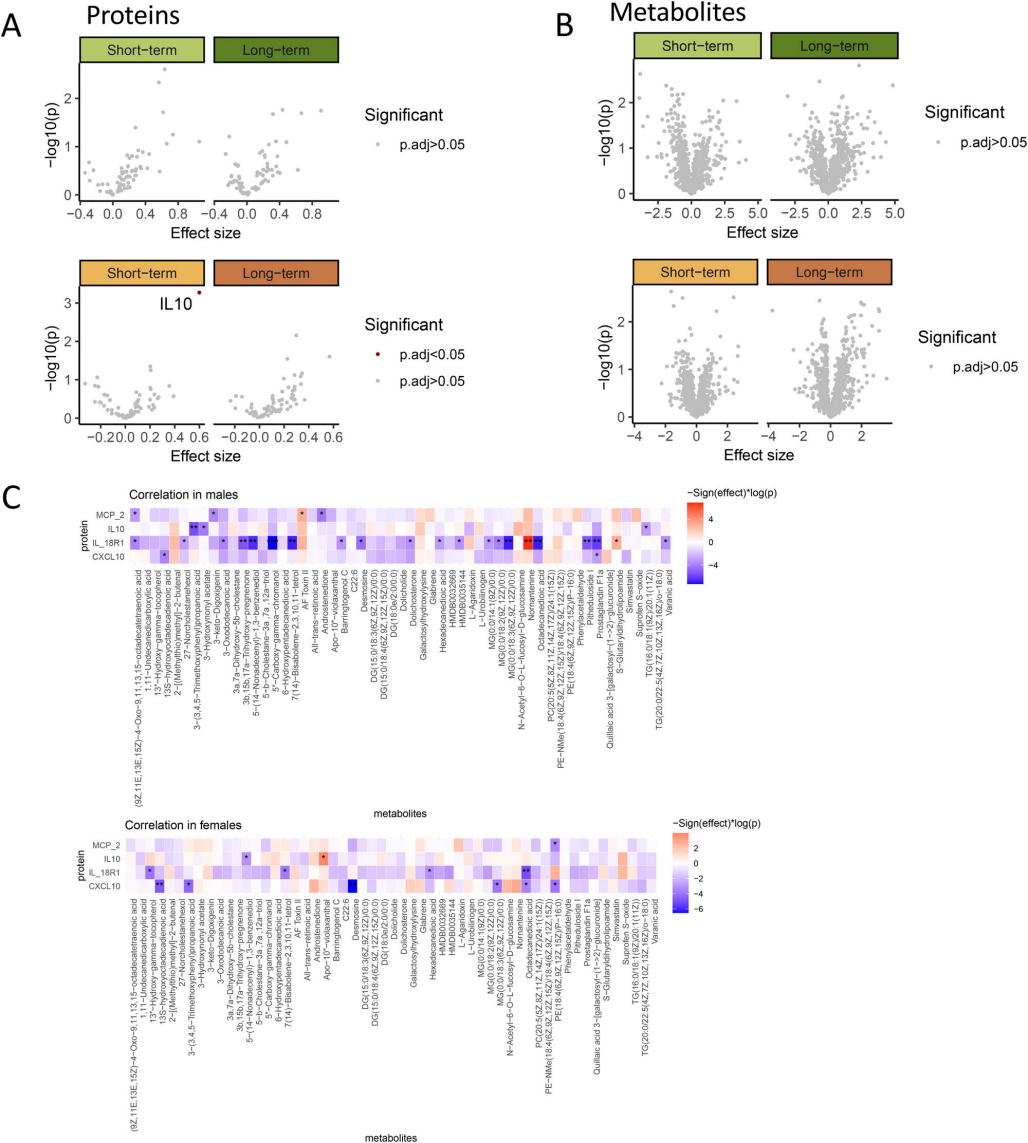
Protein (*n* = 73) and metabolite (*n* = 979) associations with vaccine efficacy identify IL‐10 as a key marker in females. (A) Volcano plot showing the effect size and significance of proteins associated with short‐term and long‐term vaccine efficacy in males (top) and females (bottom). (B) Volcano plot showing the effect size and significance of metabolites associated with short‐term and long‐term vaccine efficacy in males (top) and females (bottom). (C) The heatmap showed the correlation between the proteins and metabolites from association analysis with male short‐term vaccine efficacy in males (top) and females (bottom). *p < 0.05, **p < 0.01, ***p < 0.001.

To further explore the molecular mechanisms underlying vaccine efficacy, we analyzed the association between protein levels and vaccine efficacy in both sexes. In males, no proteins were significantly associated (*p*_FDR‐adjust_ < 0.05) with either short‐term or long‐term vaccine efficacy (Figure 4A, top). In contrast, females showed a significant positive association between IL‐10 levels and short‐term vaccine efficacy (*p*_FDR‐adjust_ < 0.05), highlighting IL‐10 as a key marker of early vaccine response in females (Figure 4A, bottom). As an anti‐inflammatory cytokine, IL‐10 is known to regulate immune responses by dampening immune cell activation and modulating cytokine production, consistent with the anti‐inflammatory transcriptional profiles observed in female high responders.

Next, we associated the metabolites with vaccine efficacy in both males and females (see details in Methods). In males, this analysis revealed no statistically significant association with short‐term or long‐term vaccine efficacy (Figure 4B, top). Similarly, in females, no metabolites showed significant associations with vaccine efficacy at either time point (Figure 4B, bottom).

To investigate the potential interplay between inflammatory proteins and metabolites that may contribute to vaccine efficacy in males and females, we illustrate the correlation between the proteins and the metabolites identified from the association analysis with short‐term vaccine efficacy in males (*p* < 0.05, Figure 4C). Notably, distinct patterns of protein‐metabolite correlation were detected between males and females. In males, a higher number of significant interactions between proteins and metabolites were observed (*p* < 0.05), particularly for IL‐18R1, which exhibited significant positive correlations with multiple metabolites, including nornantenine (Figure 4C, top). In contrast, females exhibited fewer significant protein–metabolite correlations overall (Figure 4C, bottom). Specifically, IL‐18R1 was negatively correlated with nornantenine in females, highlighting potential sex‐specific differences in how this protein interacts with metabolites. Notably, distinct patterns of protein‐metabolite correlation were detected between males and females. A significantly greater proportion of protein–metabolite correlations was observed in males compared with females (two‐proportion *z*‐test, *p* = 0.0069, Figure S4).

On the other hand, IL10 was positively correlated with beta‐D‐glucopyranosyl‐11‐hydroxyjasmonic acid in females and negatively in males. These findings indicate that the molecular mechanisms underlying vaccine efficacy may be sex‐specific, with different substances influencing the immune response in males and females (Figure S4).

### 2.6 Multi‐Omics Integration Reveals the Interplay Across Layers Impacting Vaccine Efficacy

Building on single‐omics analyses that revealed sex‐specific differences in baseline cellular and molecular factors influencing vaccine efficacy, we next employed multi‐omics factor analysis (MOFA) [29] to uncover the interconnected process across molecular layers in each sex.

**FIGURE 5:**
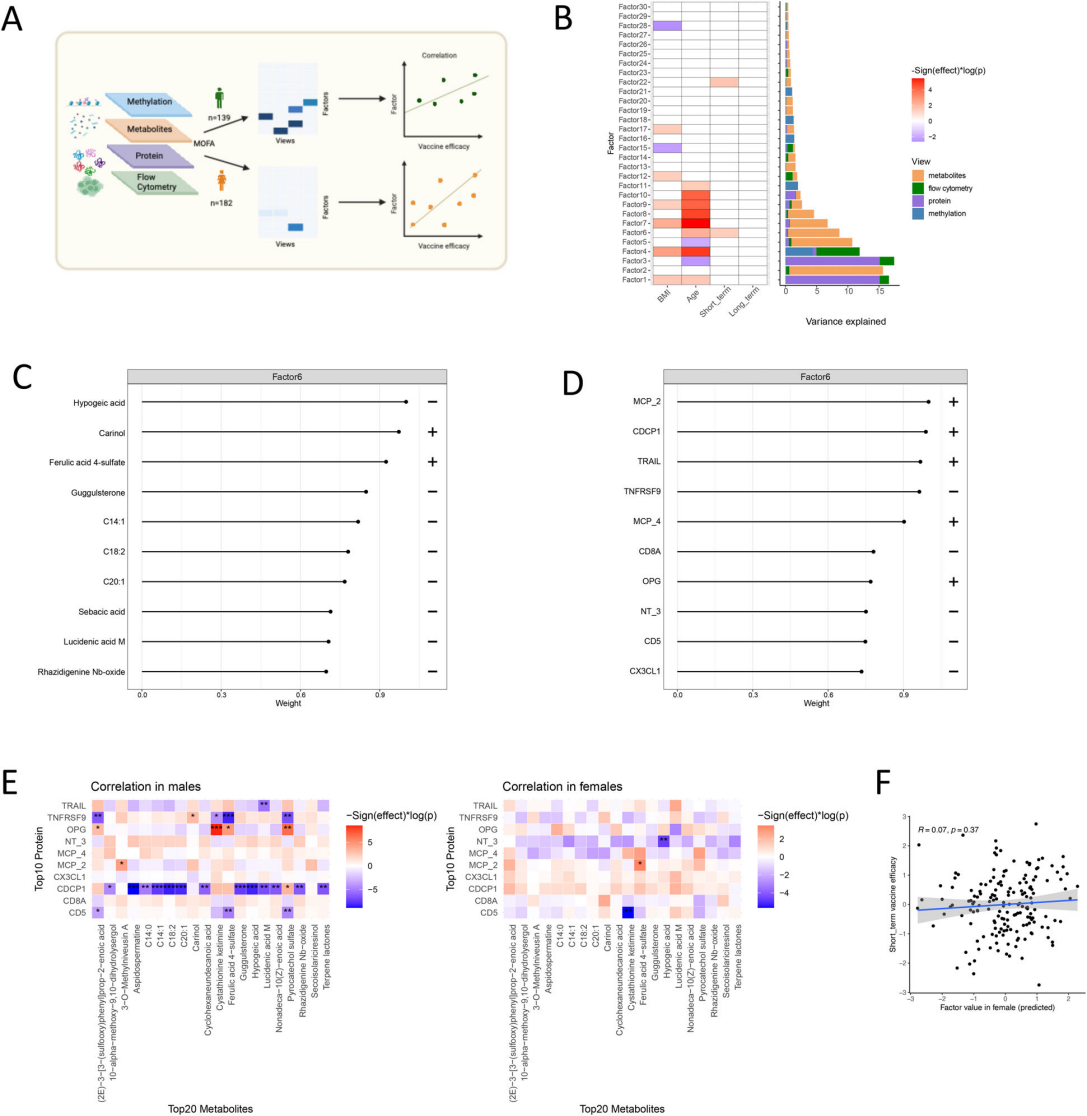
Multi‐omics integration reveals the interplay across layers impacting vaccine efficacy. (A) Overview of multi‐omics integration. (B) Left: the heatmap shows the Pearson correlation between the factor value derived by MOFA and phenotypes, including age, BMI, and vaccine efficacy in males. Right: the bar plot shows the cumulative proportion of total variance explained and the percentage of variance explained for each layer of data in males. (C) Absolute loading of metabolites and (D) proteins with the highest weights of Factor 6. The plus and minus symbols on the right indicate the sign of loading. (E) The heatmap showed the correlation between the top 10 proteins and top 20 metabolites with the highest weights of Factor 6 (left) in males and (right) in females. *p < 0.05, **p < 0.01, ***p < 0.001. (F) Scatter plot showing the correlation between predicted factor 6 value and short‐term vaccine efficacy in females. The coefficient and *p*‐value were calculated by Spearman correlation.

Our dataset included 139 male and 182 female participants, integrating 3000 of the most variable methylation CpG sites, 979 metabolites, 73 proteins, and 13 immune cell counts (Figure 5A; Figure S5A). MOFA identified 30 factors for each sex, with 13 factors in males and 9 factors in females, explaining at least 1% of the variance in one or more omics layers (Figure 5B; Figure S5B). Notably, these factors were orthogonal, capturing independent sources of variation (Figure S6A).

In males, the 30 factors collectively explained 13.7% variation in methylation data, 63.6% in metabolites data, 37.6% in protein, and 17.1% in immune cell counts (Figure S6B, left). Among these, Factor 1 predominantly captured protein‐layer variation and minor immune‐cell contributions, while Factor 6 was primarily driven by metabolites (Figure 5B). Similarly, in females, the 30 factors captured more variation in protein data (50.6%) and immune cell counts data (28.4%, Figure S6B, right).

In males, Factor 6 exhibited significant positive associations with short‐term vaccine efficacy (Figure 5B) (P<0.05, Pearson correlation). Analysis of the weights in metabolite data indicated that Factor 6 was characterized by a diverse set of bioactive molecules, including lipids, lipid‐like molecules, alkaloids, and terpenoids. Among these metabolites, Hypogeic acid, with negative weights in Factor 6, is known for its strong anti‐inflammatory effects on monocytes and macrophages [30] (Figure 5C). In addition, we observed that the proteins with the strongest positive weights on Factor 6 were proinflammation markers, including MCP‐2 [31], TRAIL [32], and CDCP1 [33] (Figure 5D). In the immune cell count layer, intermediate monocytes (CD14^+^CD16^+^), classical monocytes (CD14^++^CD16^−^), and nonclassical monocytes (CD14^++^CD16^+^) emerged as the top contributors with positive weights (Figure S7A), which aligns with our previous findings based on cell count data (Figure 1D). These findings align with our earlier observation of monocyte involvement in vaccine efficacy based on cell count data.

In females, two factors (factors 11 and 16) were significantly associated with short‐term vaccine efficacy, and two additional factors (factors 9 and 17) were linked to long‐term vaccine efficacy. Notably, Factor 9 accounted for substantial variance within the metabolite and protein layers (Figure S5B). Interestingly, this association was not observed when considering individual personal factors alone (Figure 1C). In the protein layer, SCF and TWEAK showed positive weights, while VEGFA, CSF‐1, and CDCP1 showed negative weights (Figure S7B). Interestingly, CDCP1 was negatively associated with vaccine efficacy in females, contrasting its positive association in males (Figure 5D). Metabolites with high weights in factor 9 included diverse bioactive molecules, such as alkaloids, triacylglycerols, and bile pigments (Figure S7C). In addition, the top loadings of Factor 11 in the protein layer showed positive weights for regulators such as CSF1 and HGF, and negative weights for CD40, CD244, and TNFRSF9, which were not detected in the analysis on a single layer (Figure S7D–F).

Given the sex‐specific protein–metabolite correlation patterns identified in our single‐omics analyses (Figure 4C), we next examined whether the male‐derived Factor 6 also exhibits sex‐divergent cross‐layer coordination. We calculated the correlation between the top 10 proteins and the top 20 metabolites with the largest weight in Factor 6 (Figure 5E). In males, CDCP1, which had a positive weight in Factor 6, was significantly negatively associated with the metabolites showing negative values (*p* < 0.05, Spearman correlation, Figure 5E, left). In contrast, the same analyses conducted in females demonstrated a sparser interaction network (Figure 5E, right). This indicates a coordinated interaction between proteins and metabolites that collectively shape a proinflammatory environment, thereby enhancing vaccine efficacy in males, while also underscoring the distinct mechanisms that regulate vaccine responses in a sex‐specific manner. To assess whether sex‐specific MOFA factors generalize across sexes, we cross‐applied factor loadings between males and females. First, male‐derived weights for Factor 6 were projected onto the female datasets. The resulting predicted Factor 6 values showed no association with short‐term or long‐term vaccine efficacy in females (Figure 5F; Figure S8A). Conversely, female‐derived weights for Factor 11 (short‐term efficacy) and Factor 9 (long‐term efficacy) were projected onto male datasets. Neither predicted Factor 11 nor predicted Factor 9 showed significant associations with vaccine efficacy in males (Figure S8B–E).

## 3 Discussion

In this study, we utilized a multi‐omics approach to investigate the variability in BCG vaccine response among 321 healthy individuals. To the best of our knowledge, this is the first study to integrate diverse data layers, spanning immune cell frequencies, methylation, transcription, proteins, and metabolites to uncover sex‐specific differences and pre‐vaccination immune factors that influence vaccine outcomes. Our findings revealed that the strength of BCG‐induced responses varies widely among individuals, with pronounced sex differences and pre‐vaccination immune status identified as key determinants of vaccine efficacy.

One of the most striking findings was the significant sex‐specific difference in vaccine efficacy, with males exhibiting a lower immune response than females, particularly during the early postvaccination phase. Previous studies suggest that hormonal factors, such as elevated dihydrotestosterone levels, may suppress cytokine production following BCG stimulation [34].

Furthermore, while females demonstrated a robust response characterized by adaptive immune pathways, males relied more heavily on innate immune mechanisms. These findings underscore the need to account for sex differences when evaluating vaccine responses and developing immunization strategies.

Multiple omics layers consistently pointed to the central role of pro‐inflammatory pathways in driving vaccine efficacy. Key signaling pathways, such as p38 MAPK [28] and mTOR pathways, were activated in males with higher vaccine response. These pathways are critical for cytokine production, innate immune activation, and affect cell recruitment. In contrast, females exhibited antigen presentation and processing pathways as the dominant contributors to vaccine efficacy. Factors such as IL‐10 and SCF were positively associated with vaccine efficacy, indicating their roles in modulating inflammation and supporting adaptive immunity. The enrichment of MHC class II activation further emphasized the importance of antigen presentation in promoting robust vaccine responses in females.

Baseline immune states emerged as a critical determinant of vaccine efficacy. Individuals with low baseline cytokine production, particularly IFN‐γ, exhibited a significant enhancement in immune responses postvaccination. Conversely, individuals with higher pre‐vaccination IFN‐γ levels showed minimal improvement, consistent with findings in other live viral vaccines such as yellow fever, smallpox, and dengue vaccine [35]. It suggests that elevated interferon activity may suppress pathogen replication and antigen presentation, thereby reducing vaccine effectiveness.

BMI was another host factor associated with vaccine cellular responses, but this association was exclusive to males. This sex‐specific pattern is consistent with previous studies showing that obesity modifies vaccine‐induced antibody responses predominantly in men, but not women, likely due to sex differences in adipose tissue distribution, and estrogen‐enhanced adaptive immunity may buffer such BMI‐related effects [36, 37, 38, 39]. While these findings align with prior reports on obesity and inflammation, the predominantly normal BMI range in our cohort limits broader generalization. Future studies in more diverse populations are needed to better understand the role of BMI in vaccine responses.

Our data suggest that males and females leverage distinct immune strategies in response to BCG vaccination [40]. While males rely on innate pro‐inflammatory responses, females emphasize antigen presentation and adaptive immunity. These findings highlight the potential for targeted interventions, such as enhancing antigen presentation pathways, to improve vaccine efficacy. For instance, previous studies have shown that rapamycin, which induces autophagy and enhances antigen presentation, can improve vaccine outcomes, suggesting a viable strategy for bridging the efficacy gap [41, 42].

This study has several limitations. First, the cohort was limited to healthy European individuals; most of them were young with normal BMI, making them less representative of the general population. Second, genetic analysis [43] was not included due to the lack of statistical power for exploring sex‐specific genetic differences. Future studies should address these omissions by including more diverse populations, controlling for environmental factors, and integrating genetic data.

## 4 Methods

### 4.1 Study Cohort

In the 300BCG cohort, 321 healthy Western European ancestry individuals were recruited at the Radboud University Medical Center between April 2017 and June 2018. The group included 182 females and 139 males. After obtaining informed consent, a standard dose of 0.1 mL BCG (Bulgaria strain, Intervax) was intradermally injected into the individual's upper arm. EDTA blood samples were collected at three time points: before vaccination, 14 days, and 90 days after vaccination. The participants were excluded if they had a history of BCG vaccination or tuberculosis infection, a medical history of immunodeficiency or other chronic diseases, used systemic medication except for oral contraceptives or acetaminophen, taken antibiotics in the 3 months before enrollment, received any vaccination within three months before recruitment, experienced acute febrile illness 4 weeks before inclusion, or were pregnant.

### 4.2 Measurement of Cytokine Production

Peripheral blood mononuclear cells (PBMCs) were collected at three‐time points: before vaccination (Day 0), early postvaccination phase (Day 14), and later postvaccination phase (Day 90). In a total volume of 200 µL/well, 5 × 10^5^ PBMCs were cultured in round‐bottom 96‐well plates (Greiner) and stimulated with heat‐killed *M. tuberculosis* H37Rv (5 µg/mL). The IFN‐γ concentrations (Luminex, Thermo Fisher) were measured after being stimulated by *M. tuberculosis* for 7 days. Cytokine production in response to *M. tuberculosis* was evaluated at these three‐time points. The fold change of IFN‐γ concentrations from baseline to Day 14 (short‐term vaccine efficacy) and Day 90 (long‐term vaccine efficacy) was used as a measure of BCG vaccine efficacy.

### 4.3 Measurement of Immune Cell Frequencies

Venous blood was collected from the cubital vein into 10 mL EDTA (Monoject). A total of half a million leukocytes were analyzed for surface markers using the Navios flow cytometer (Beckman Coulter). Cells were stained for 20 min at room temperature in the dark with the fluorochrome‐conjugated monoclonal antibodies. After flow cytometry analysis, the cell‐type proportions for monocytes, neutrophils, and lymphocytes among the PBMCs were calculated based on fluorescence‐activated cell sorting (FACS) analysis of whole blood cells.

### 4.4 Measurement of Methylation and Analysis

Blood DNA was isolated by QIAamp DNA micro kit (Qiagen Benelux BV, Venlo, the Netherlands). DNA methylation was profiled using whole‐blood samples collected at baseline before BCG vaccination. Genome‐wide methylation levels were assayed with the Illumina Infinium MethylationEPIC BeadChip (∼850,000 CpG sites). QC steps are described in the previous study [5]. After Quality Control, a total of 751,564 probes and 284 samples were retained for analysis. Methylation levels were converted into M values. We set extreme outliers as missing, which were identified using the Tukey method (<1st quartile‐3´ IQR; >3rd quartile+3´ IQR). The identified significant CpG sites (*p*_FDR‐adjusted_ < 0.05) were annotated to upstream and downstream genes by the GREAT annotation tool [44].

### 4.5 Measurement of Metabolites and Quality Control

Nontargeted metabolite levels were measured before BCG vaccination and annotated by the General Metabolics using flow injection time‐of‐flight mass (TOF‐M) spectrometry [45]. In total, 1607 endogenous metabolites were annotated according to the Human Metabolites Database (HMDB) [46] based on the ion's chemical formula. Features with an annotation score <70 were excluded; 1373 features remained. Subsequently, we manually cross‐referenced these metabolites with the DrugBank database [47] to verify whether they were related to drugs. Metabolites associated with medications or other xenobiotics were then removed from the analysis. After quality control, 979 metabolites were retained for downstream analyses.

### 4.6 Measurement of Circulating Inflammatory Markers

Circulating plasma inflammatory proteins were measured in plasma using the Olink Proteomics AB Inflammation Panel [48], using a Proceek Multiplex proximity extension assay. Signals were processed with the Olink pipeline and expressed as log_2_ normalized protein expression (NPX) to minimize interassay variation. Samples that failed Olink QC and proteins with >25% measurements at or below the lower limit of detection were excluded. After QC, 73 proteins remained and were used for analysis.

### 4.7 Single‐Cell Transcriptome Analysis

To investigate the profile of different responders at single‐cell resolution. Single‐cell transcriptome data at baseline were extracted from the previous study [9]. Based on the median value of the fold change of IFN‐γ concentrations between Day 14 and Day 0 across all 321 participants, individuals were classified into high responders (above median) and low responders (below median). Because one of the individuals lacked the measurement of IFN‐γ concentration at 14 days after vaccination, it was excluded from downstream analysis. For each main cell type, we identified differentially expressed gene‐analysis between high responders and low responders using FindMarkers function in the Seurat package (v4.0.0) [49]. The genes were expressed in at least 10% of cells, and those with p.adj (Bonferroni post hoc correction) < 0.05 were considered significant. After identifying the DEGs, we clustered the average of log_2_ fold change profiles of significance, considering five main cell types. Before clustering, insignificant values were set to 0. We performed *k*‐means clustering with *K* = 12 and default parameters in the ComplexHeatmap package (v2.7.7) [50]. Considering variable *K* values, splitting or merging clusters at the margin, we picked *K* = 12 as the lowest value that yielded the cluster of monocyte‐specific upregulated genes. Pathway enrichment of significant genes was performed using the ConsensusPathDB‐human platform [51]. The R package UCell (v2.0.1) was used to calculate the gene sets activity score with default parameters, and *p*‐value in UCell score comparisons were calculated using Wilcoxon's rank‐sum test.

To directly evaluate sex‐specific transcriptional effects, pseudobulk differential‐expression models were fitted using the design ∼ age + batch + sex + responder + sex:responder.

The interaction term (sex: responder) was used to infer sex‐dependent differences in high‐ versus low‐responder contrasts. Genes were ranked by the statistic of the interaction term for pathway enrichment using Reactome GSEA.

### 4.8 Integration of Multi‐Omics Data

The single‐cell dataset included only 38 individuals (17 males), limiting the power for integrated analysis. Therefore, we focused on four layers with a larger sample size (*n* = 139 males): metabolites, proteins, methylation, and immune cell proportion. To prevent overrepresentation of large modalities, we reduced the dimensionality of the methylation data (up to 750,000 CpG sites). We selected the top 3000 methylation CpG sites with the highest variance. Additionally, count‐based data, such as immune cell proportions, were normalized using a natural‐log transformation. This integrated analysis incorporated 3000 CpG sites, 979 metabolites, 73 proteins, and 18 immune cell proportions of males at baseline.

To achieve unsupervised integration of the four data layers, we employed MOFA2 [29] in slow convergence mode. For the MOFA training options, we set the number of factors to 30 and also scaled the data within each view to ensure comparability. We then conducted Pearson correlation tests between factor values derived by MOFA and phenotypes (BMI, age, short‐term vaccine efficacy, and long‐term vaccine efficacy). Based on the distribution of the scaled weights of Factor 6, metabolites with absolute weights > 0.1 were considered for the enrichment analysis. For replication in females, we extracted the scaled weights of Factor 6 and applied them to the female dataset using the following formula: factor value = vector (weights) × matrix (data).

### 4.9 Statistical Analyses

To estimate the effects of host factors on vaccine efficacy, Spearman correlation analysis was performed on the fold change of IFN‐γ concentrations (Day14/Day0, Day90/Day0) and host factors, including BMI, age, and baseline IFN‐γ concentration.

To formally test whether the association between BMI and vaccine efficacy differed by sex, we fitted linear regression models including BMI, sex, and their interaction term as follows:

Vaccineefficacy∼Sex+BMI+BMI×Sex+Age.

The coefficient of the Sex × BMI interaction term was used to assess sex‐specific differences in the BMI–efficacy relationship.

Besides, to estimate the effects of personal immune profiles at baseline on vaccine efficacy, we assessed the association between cytokine production changes and immune cell proportions, metabolites, and proteins by fitting the linear model adjusting for age, separately. Associations between baseline immune cell frequencies and vaccine efficacy were first examined in sex‐stratified analyses using Spearman rank correlations. To formally assess sex differences in these associations, we fitted linear models including an interaction between cell proportion and sex while adjusting for age:

$$\begin{array}{ccc} \mathrm{Vaccine}\;\mathrm{efficacy} & ∼ & \mathrm{Cell}\_\mathrm{proportion}+\mathrm{Sex}+\mathrm{Cell}\_\mathrm{proportion}\\ & & \times \;\mathrm{Sex}+\mathrm{Age}. \end{array}$$

The *p*‐value of the Cell_proportion × sex interaction term was used to test whether the cell–efficacy association differed between males and females.

We also assessed the association between fold change of IFN‐γ and DNA methylation levels using a robust linear regression model, correcting for age, sample plate, and estimated proportions of CD8 T cells, CD4 T cells, NK cells, B cells, monocytes, and neutrophils using IWLS iterations of 200. The coefficients derived from the linear regression model represented the association effect. Within sex‐significant CpG sets, sex differences in effect sizes were assessed using paired Wilcoxon signed‐rank tests on Δ_abs_ = |Effect_size_emale_|−|Effect_size_male_| (male‐significant: one‐sided H1: Δ_abs_ < 0; female‐significant: H1: Δ_abs_ > 0), and binomial tests of magnitude dominance Pr(|Effect_size_male_|>|Effect_size_female_|) >0.5 or Pr(|Effect_size_male_|<|Effect_size_female_|) >0.5.

Network density was computed as D = E/U with a common edge universe U = n(protein) ×m(metabolites). Edges were retained using a fixed association threshold with *p* < 0.05 for topology quantification. Two‐proportion *z*‐test inference pertains to global density differences.

Adjusted *p*‐values were calculated by Benjamini and Hochberg's false‐discovery rate (*p*_FDR‐adjusted_) method for multiple testing. A *p*‐value of less than 0.05 was considered significant.

## Author Contributions

Y.L., M.G.N., and C.J.X. conceptualized and designed the study. Q.Z. performed the data analysis supervised by C.J.X., Y.L., and M.G.N., S.J.C.F.M.M., V.A.C.M.K., L.C.J.d.B., and V.P.M. recruited the participants and collected the biological material. J.F. and X.L. helped with part of the data analysis. L.Z. validated the results. Q.Z., C.J.X., Y.L., and M.G.N. wrote the manuscript. All authors reviewed and approved the manuscript.

## Funding

C.J.X. is supported by the Lower Saxony MWK Sprung Fund (19777006) and Deutsche Forschungsgemeinschaft (DFG) Fund (497673685). Y.L. is supported by an ERC Starting Grant (948207). M.G.N. is supported by an ERC Advanced Grant (833247) and a Spinoza Grant of the Netherlands Organization for Scientific Research. Q.Z. was supported by a grant from the China Scholarship Council.

## Ethics Approval and Consent to Participate

The study was approved by the Arnhem‐Nijmegen medical ethical committee (NL58553.091.16). Written informed consent was obtained before any research procedure was initiated. All studies were performed in accordance with the Declaration of Helsinki.

## Conflicts of Interest

The authors declare no conflicts of interest.

## Clinical Trial Number

Not applicable.

## Consent for Publication

Not applicable.

## Supporting information




**Supporting File 1**: eji70144‐sup‐0001‐tableS1.xlsx.

**Supporting File 2**: eji70144‐sup‐0002‐tableS2.xlsx.

**Supporting File 3**: eji70144‐sup‐0003‐figureS1‐S8.pdf.

**Supporting File 4**: eji70144‐sup‐0004‐CaptionS1‐S2.docx.

## Data Availability

DNA methylation data have been deposited at the European Genome‐phenome Archive (EGA), which is hosted by the EBI and the CRG, under accession number EGAS00001007498. Single‐cell RNA‐seq data has been deposited at the European Genome‐phenome Archive (EGA) under accession number EGAS00001006990. The code generated to analyze the data is freely available on Github (https://github.com/CiiM‐Bioinformatics‐group/BCG_vaccine_efficacy).
