# Supplementary material for: Multi‐Omics Analysis Reveals Sex‐Specific Signatures for BCG Vaccine Efficacy
Source: Eur J Immunol. 2026 Feb 12;56(2):e70144. doi: 10.1002/eji.70144 (PMC12896084; doi:10.1002/eji.70144)
Supplement: Supplementary file 3 — Supporting File 3: eji70144‐sup‐0003‐figureS1‐S8.pdf. [file EJI-56-e70144-s003.pdf]

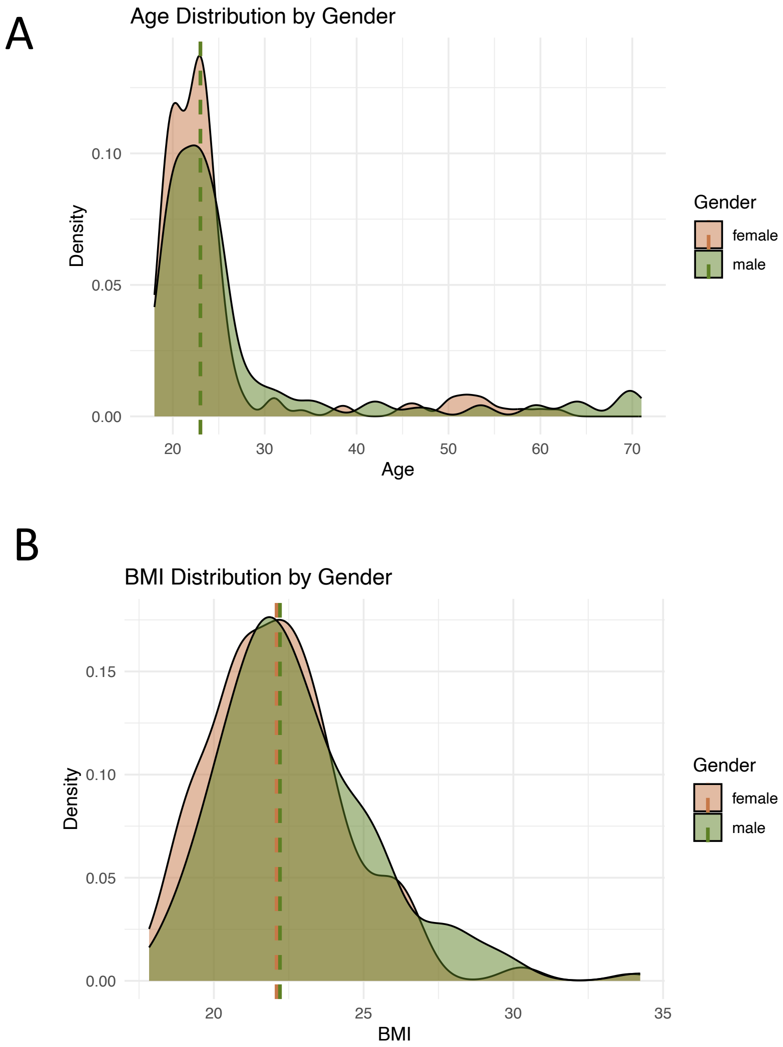

Fig. S1 Overview of the cohort. The density plot represents the **(A)** age and **(B)** BMI distribution of the cohort, stratified by gender. The orange shaded area indicates females, and the green shaded area represents males. The vertical dashed lines indicate the median age for each gender (orange for females, green for males). Most participants were under the age of 30 and the BMI of 25.

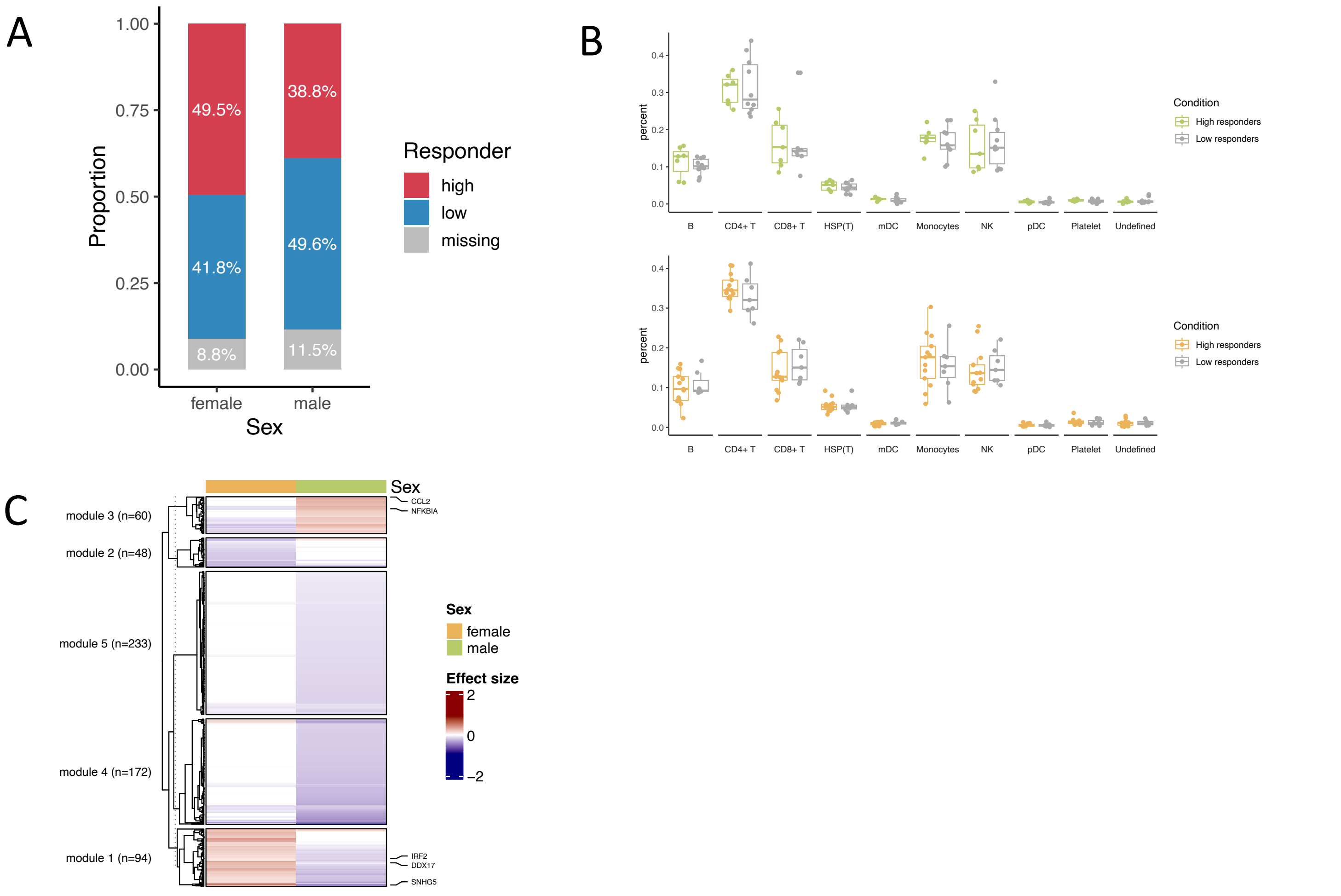

Fig. S2 Comparison between high and low vaccine responders in both sexes. (A) Proportions of high, low, and missing vaccine responders among females and males in the full cohort (n = 321), defined using the median Day14/Day0 IFN-γ fold change. No significant difference in responder distribution was observed between sexes (Fisher's exact test, p = 0.09). **(B)** Top: Boxplots depict the frequencies of various immune cell populations between high and low responders in (**top**) males and (**bottom**) females. Data are stratified by the "high responders" (male: green, female: orange) and "low responders" (gray) conditions. **(C**) Heatmap of effect sizes for DEGs in monocytes from each sex. Rows represent DEGs, and columns show effect sizes in males and females. Colors indicate effect size magnitude. The n means the number of DEGs.

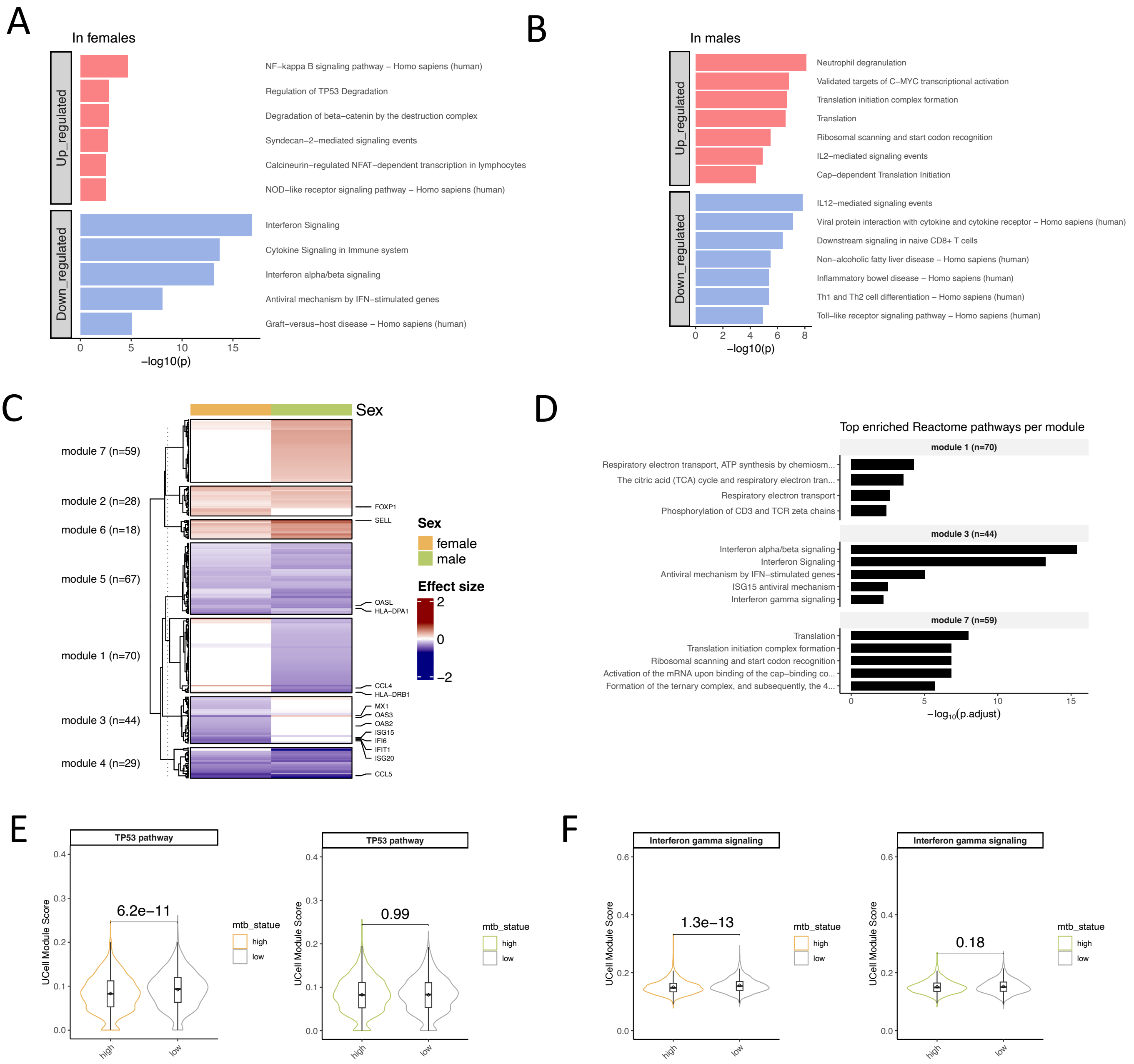

Fig. S3 Comparison between high and low vaccine responders in CD8⁺ T cells. (A) The bar chart shows significantly up-regulated and down-regulated pathways based on pathway enrichment analysis from differentially expressed genes in females and **(B)** in males. Bars show the top enriched pathways ranked by –$\log_{10}$(p-value). **(C)** Heatmap of effect sizes for DEGs in CD8+ T cells from each sex. Rows represent DEGs, and columns show effect sizes in males and females. Colors indicate effect size magnitude. **(D)** Reactome pathway enrichment analysis of module 1 (female-downregulated), module 3 (female-specific downregulated), and module 7 (male-specific upregulated) DEGs in CD8$^+$ T cells from Fig. S3C. **(E-F)** The gene set score of 'TP53 Regulates Transcription of Genes Involved in Cytochrome C Release' and 'Interferon gamma signaling' pathway in CD8$^+$ T cells in females (left) and males (right). The violin plots are color-coded based on high and low responders. Boxplots summarize the distribution of gene set scores, with the horizontal line indicating the median. Wilcoxon's rank-sum test was applied to compare the gene set scores between high responders and low responders.

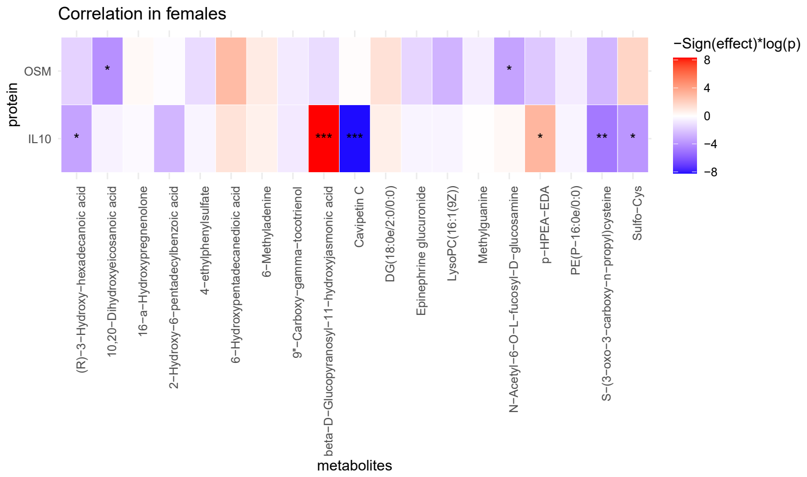

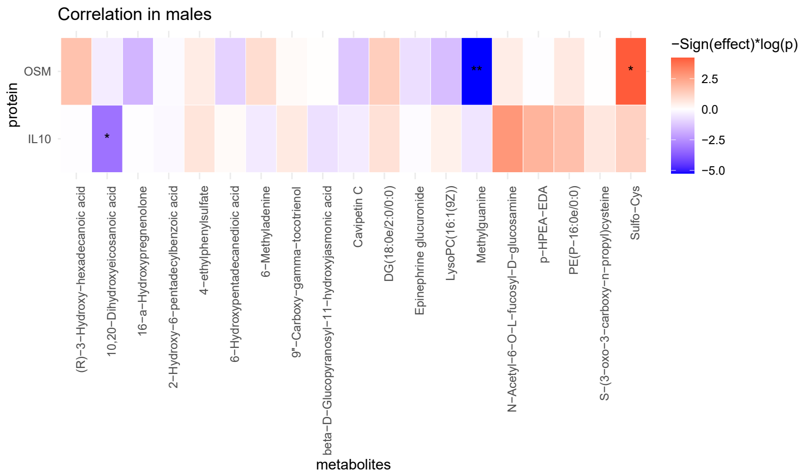

Fig. S4 Correlation between proteins and metabolites identified in association with vaccine efficacy for females. The heatmaps illustrate the correlations between selected proteins and metabolites significantly associated with vaccine efficacy in females($p<0.05$), separated by sex (top panel: females, bottom panel: males). Spearman correlations were calculated for each protein-metabolite pair. Each cell color represents the correlation coefficient, with blue indicating negative correlations and red indicating positive correlations. The color intensity corresponds to the strength of the correlation. Asterisks represent the significance levels: *$p < 0.05$, **$p < 0.01$, and ***$p < 0.001$.

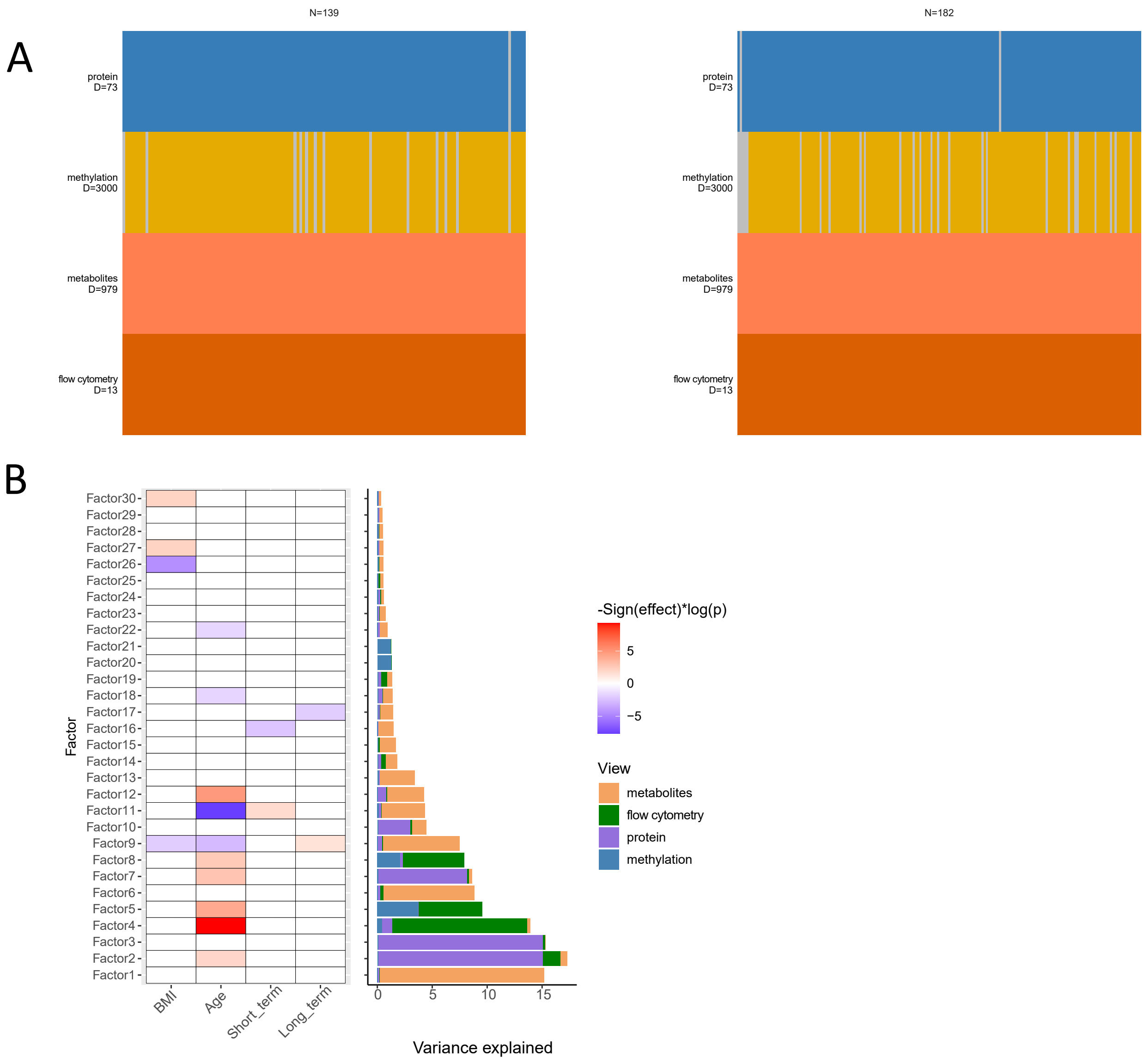

Fig. S5 Multi-omics integration by MOFA. (A) Study overview and data types of males (left) and females (right). Data modalities are shown in different rows (D = number of features) and samples (N) in columns, with missing samples shown using grey bars. **(B)** The heatmap shows the Pearson correlation between the factor value derived by MOFA and phenotypes including age, BMI, and vaccine efficacy in females. The bar plot shows the cumulative proportion of total variance explained and the percentage of variance explained for each layer of data.

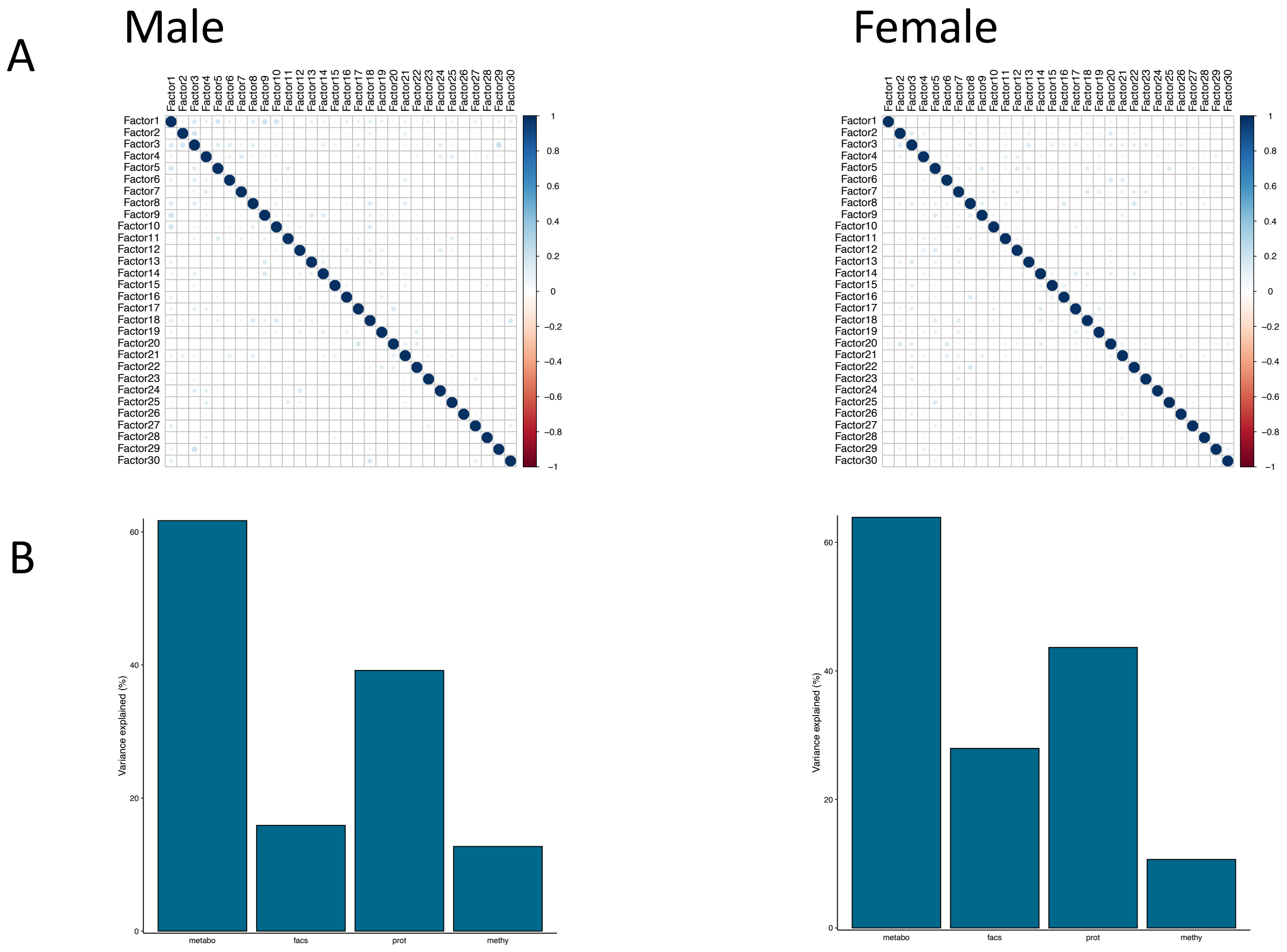

Fig. S6 Overview of factors identified by MOFA. (A) Pearson correlation between factors (left: male, right: female). **(B)** Global explained variance for each of the data modalities has been represented as a bar chart (left: male, right: female).

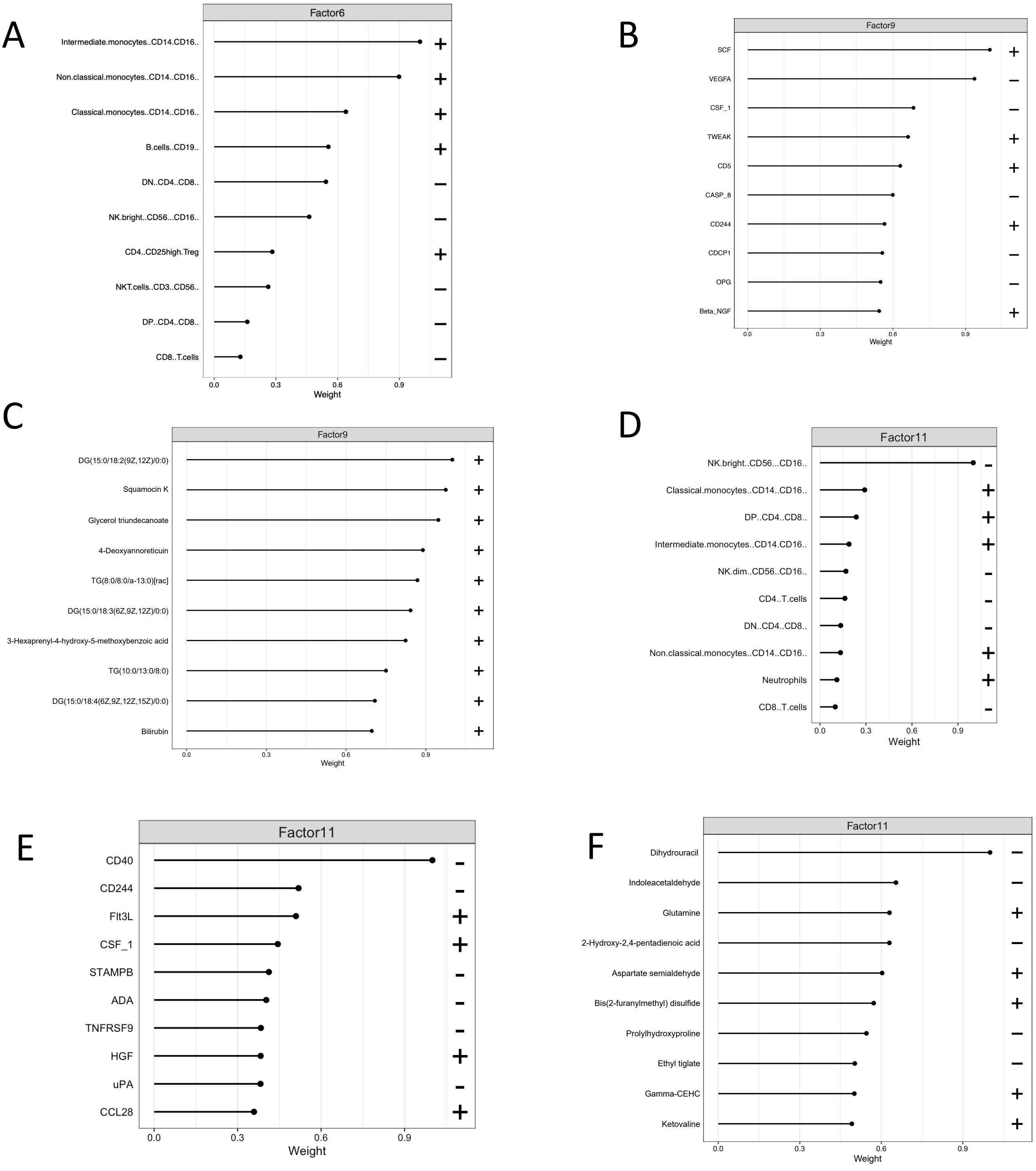

Fig. S7 Characterization of the inferred factors. (A) Absolute loading of cell proportion with the highest weights of Factor 6 in males. **(B-C)** Absolute loading of proteins and metabolites with the highest weights of Factor 9 in females. **(E-F)** Absolute loading of cell proportion, proteins and metabolites with the highest weights of Factor 11 in females. Plus or minus symbols on the right indicate the sign of the loading.

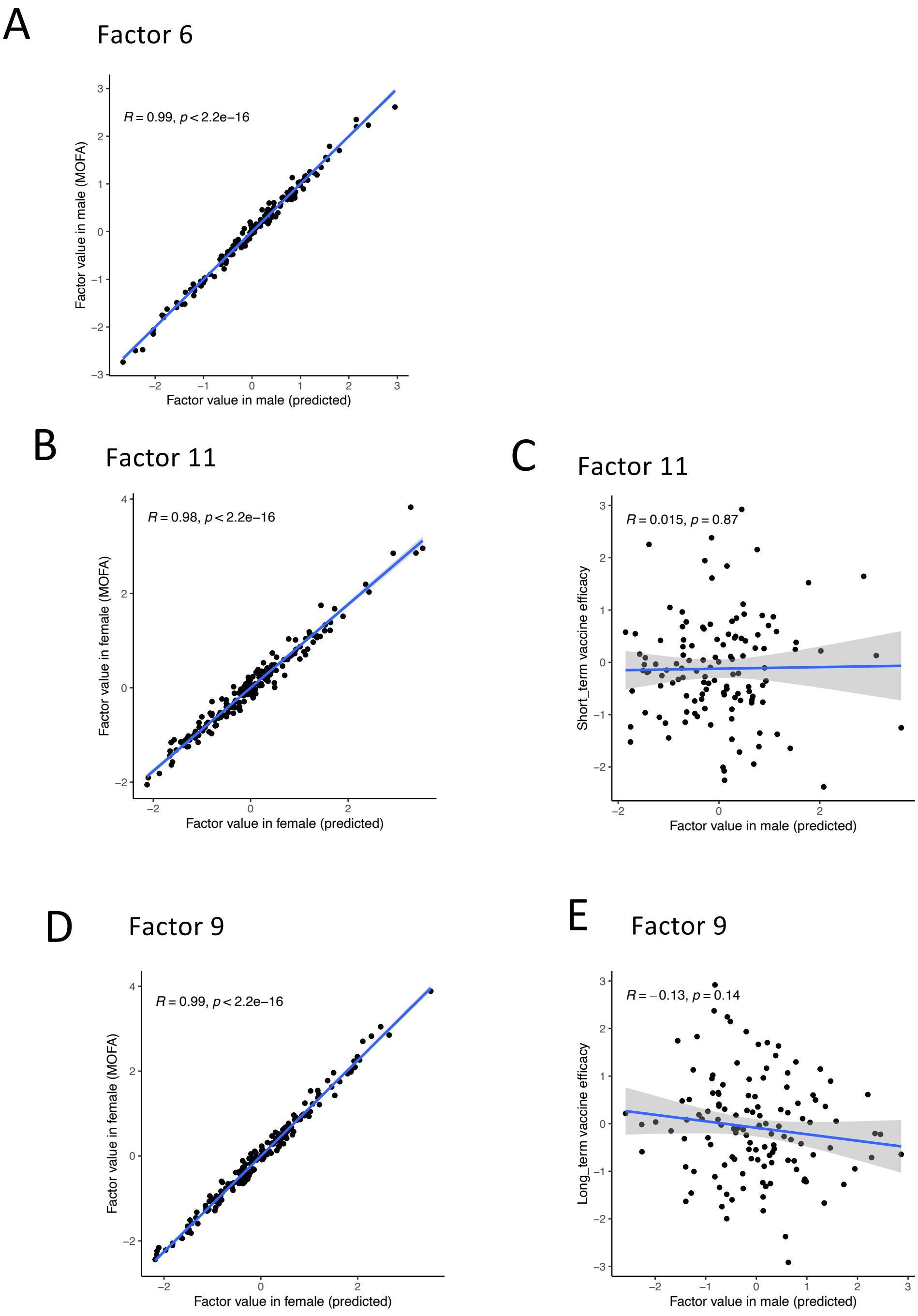

Fig. S8 Characterization of the inferred factors. (A) Correlation between predicted and MOFA-derived Factor 6 values in males. **(B)** Correlation between predicted and MOFA-derived Factor 11 values in females. **(C)** Correlation between predicted Factor 11 values and long-term vaccine efficacy in males. **(D)** Correlation between predicted and MOFA-derived Factor 9 values in females. **(E)** Correlation between predicted Factor 9 values and short-term vaccine efficacy in males. The coefficient and P value were calculated by Spearman correlation.
