# Supplementary material for: Multi‐Omics Analysis Reveals Sex‐Specific Signatures for BCG Vaccine Efficacy
Source: Eur J Immunol. 2026 Feb 12;56(2):e70144. doi: 10.1002/eji.70144 (PMC12896084; doi:10.1002/eji.70144)
Supplement: Supplementary file 4 — Supporting File 4: eji70144‐sup‐0004‐CaptionS1‐S2.docx. [file EJI-56-e70144-s002.docx]

**Supplementary Tables**

**Table S1. Over-representation analysis of monocyte-specific modules**

**Table S2. GSEA based on interaction-term statistics on the pseudobulk level**
